# Supplementary material for: LncRNA TCONS_00323213 Promotes Myogenic Differentiation by Interacting with PKNOX2 to Upregulate MyoG in Porcine Satellite Cells
Source: Int J Mol Sci. 2023 Apr 5;24(7):6773. doi: 10.3390/ijms24076773 (PMC10094759; doi:10.3390/ijms24076773)
Supplement: Supplementary file 1 [file ijms-24-06773-s001.zip › Figure S1.pdf]

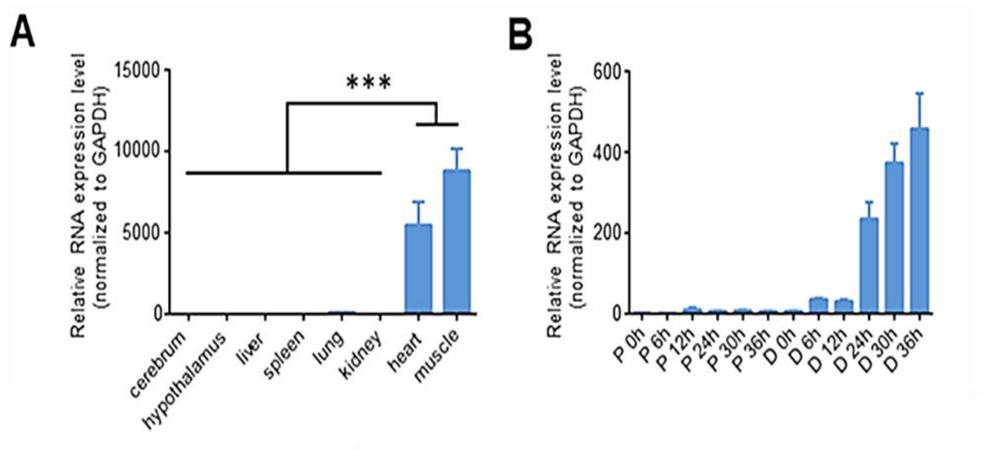

Figure S1. *TCONS_00323213* is a muscle-specific expression and closely relative to muscle differentiation lncRNA. (A)Real-time quantitative PCR analysis of *TCONS_00323213* expression in 1-week old male pig tissues. (B) Real-time quantitative PCR analysis of TCONS_00323213 expression during proliferation and differentiation state in primary pig skeletal muscle satellite cells, respectively. Letter P and D represent cells cultured in growth and differentiation medium, respectively. Mean values ± SD, n = 3. *$p < 0.05$, **$p < 0.01$, ***$p < 0.001$.*
