# Supplementary material for: LncRNA TCONS_00323213 Promotes Myogenic Differentiation by Interacting with PKNOX2 to Upregulate MyoG in Porcine Satellite Cells
Source: Int J Mol Sci. 2023 Apr 5;24(7):6773. doi: 10.3390/ijms24076773 (PMC10094759; doi:10.3390/ijms24076773)
Supplement: Supplementary file 1 [file ijms-24-06773-s001.zip › Figure S2.pdf]

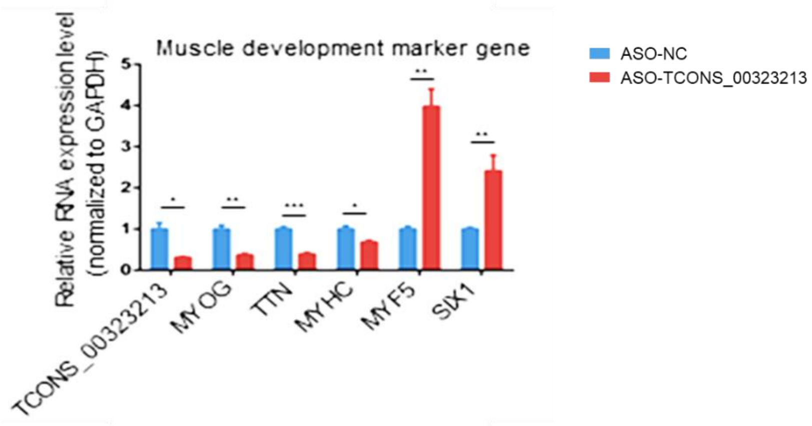

Figure S2. Real-time quantitative PCR analysis of marker genes expression level in differentiated 36 h after ASO-*TCONS_00323213* transfected in PSCs. Mean values ± SD, n = 3. $^*p < 0.05$, $^{**}p < 0.01$, $^{***}p < 0.001$.
